# Physicochemical Niche Conditions and Mechanosensing by Osteocytes and Myocytes

**DOI:** 10.1007/s11914-019-00522-0

**Published:** 2019-08-19

**Authors:** Jianfeng Jin, Astrid D. Bakker, Gang Wu, Jenneke Klein-Nulend, Richard T. Jaspers

**Affiliations:** 1 Department of Oral Cell Biology, Academic Centre for Dentistry Amsterdam (ACTA), University of Amsterdam and Vrije Universiteit Amsterdam, Amsterdam Movement Sciences, Amsterdam, The Netherlands; 2 Department of Oral Implantology and Prosthetic Dentistry, Academic Centre for Dentistry Amsterdam (ACTA), University of Amsterdam and Vrije Universiteit Amsterdam, Amsterdam Movement Sciences, Amsterdam, The Netherlands; 3 Laboratory for Myology, Faculty of Behavioral and Movement Sciences, Vrije Universiteit Amsterdam, Amsterdam Movement Sciences, De Boelelaan 1108, 1081 HZ Amsterdam, The Netherlands

**Keywords:** Physicochemical niche conditions, Mechanical stimuli, Mechanosensing, Mechanotransduction, Osteocyte, Myoblast

## Abstract

**Purpose of Review:**

Bone and muscle mass increase in response to mechanical loading and biochemical cues. Bone-forming osteoblasts differentiate into early osteocytes which ultimately mature into late osteocytes encapsulated in stiff calcified matrix. Increased muscle mass originates from muscle stem cells (MuSCs) enclosed between their plasma membrane and basal lamina. Stem cell fate and function are strongly determined by physical and chemical properties of their microenvironment, i.e., the cell niche.

**Recent Findings:**

The cellular niche is a three-dimensional structure consisting of extracellular matrix components, signaling molecules, and/or other cells. Via mechanical interaction with their niche, osteocytes and MuSCs are subjected to mechanical loads causing deformations of membrane, cytoskeleton, and/or nucleus, which elicit biochemical responses and secretion of signaling molecules into the niche. The latter may modulate metabolism, morphology, and mechanosensitivity of the secreting cells, or signal to neighboring cells and cells at a distance. Little is known about how mechanical loading of bone and muscle tissue affects osteocytes and MuSCs within their niches.

**Summary:**

This review provides an overview of physicochemical niche conditions of (early) osteocytes and MuSCs and how these are sensed and determine cell fate and function. Moreover, we discuss how state-of-the-art imaging techniques may enhance our understanding of these conditions and mechanisms.

## Introduction

Development and maintenance of bone and muscle mass are fundamental processes in mammalian physiology, since the musculoskeletal system allows movement and provides support and protection for vital organs [1]. The musculoskeletal system does not only have an essential mechanical function, muscle and bone also provide a systemic supply of metabolites and signaling molecules. Bone serves as an ion pool for maintaining serum levels of Ca^2+^, Mg^2+^, and other physiological key elements and produces active endocrine products such as FGF23 and osteocalcin [2–4]. Muscle, as a consumer and storage site for glucose and amino acids, secretes various myokines (myostatin, IL-6, IL-7, IL-8, CXCL-1, LIF, IL-15, Akt1, FGF-21, BDNF, calprotectin, erythropoietin, and IL-4), thereby affecting metabolism in other tissues such as bone [5, 6]. In terms of the far-reaching functions of these tissues in human health, a detailed understanding of the conditions that influence bone and muscle health is important, particularly those conditions that significantly decrease bone and muscle mass during prenatal or postnatal development or in adults as a consequence of aging, as well as those conditions that prevent loss of bone and muscle mass. Such knowledge will help to maintain physical mobility, decrease the risk of human injury and metabolic diseases, and ultimately improve the quality of life and life expectancy [1].

Bone and muscle mass are influenced by several factors, including nutrition, hormones, genetics, growth factors, and mechanical stimuli [7, 8]. Muscle and bone are not the only tissues sensitive to mechanical loading. Moreover, bone and muscle cells have few similarities and reside in very different niche conditions. However, close communication and/or interaction between bone and muscle is very important. In this review, we focus on mechanical stimuli as influential factor, since changes in mechanical stimuli are incredibly potent regulators of both human bone and muscle mass. It is well-known that increased mechanical stimulation of skeletal muscle leads to an increase in muscle fiber cross-sectional area (i.e., hypertrophy), while reduced mechanical stimulation results in rapid muscle atrophy [9, 10]. At the critical stages of bone development and bone formation in response to injury, mechanical stimuli increase bone mass and/or reinforcement of bone tissue, bone mineral accrual, and bone strength. In contrast, reduced mechanical stimulation of mature bone and loss of mechanical stimuli at critical stages of bone growth, cause loss of bone mass, bone mineral accrual, and bone strength [11, 12•]. Importantly, the maintenance and development of bone mass is affected by skeletal muscle-derived mechanical stimuli [13]. Physical exercise might thus provide a safe and relatively cheap therapeutic intervention to maintain or enhance bone and muscle mass and thereby overall health and fitness, at least in healthy individuals. Unfortunately, real progress is hampered because the knowledge on the exact cellular processes activated by physical stimuli seems fragmented. There is relatively little knowledge on how these cellular processes are affected by aging and disease. For a better overall understanding of mechanotransduction by musculoskeletal-derived cells in disease and aging, more studies should focus not only on fundamental cellular responses to mechanical pertubations of musculoskeletal-derived cells in isolation but also in relation to the environment in which the mechanosensitive cell types reside.

Mechanical loading of bone and muscle causes cellular, cytoskeletal, and nuclear deformation [14] and stimulates transmembrane molecules acting as mechanosensors [12•, 15•]. The transmembrane molecules available to function for a given cell depend on the immediate environment of said cell, i.e., the presence of different types of neighboring cells contacting the mechanosensitive cell and the composition of the extracellular matrix (ECM). In addition, the availability of oxygen, signaling molecules, and physical properties of the ECM are all likely to affect the ability of musculoskeletal-derived cells to sense and respond to mechanical cues. These far-reaching consequences of the exact chemical, physical, and cellular composition of the direct environment in which a specific cell type resides for the ability of said cell type to function has been widely accepted in the field of stem cell research, where it is known as “niche condition.” This review provides a literature-based overview of the physicochemical niche conditions of (pre)-osteocytes and myocytes and how these niche conditions affect the manner in which mechanical cues are sensed, thereby determining cell fate and function. We focus on (pre)-osteocytes since these are the most frequently studied cells in relation to mechanosensing and seem to be ideally situated in an environment that allows amplification of mechanical signals. In addition, osteocytes are extremely potent producers of paracrine and endocrine signaling molecules, making them orchestrators of bone mass and bone architecture [16]. For muscle, we focus on the muscle stem cell (MuSC), also referred as satellite cell, as this is the mono-nuclear muscle precursor cell, required for myofiber regeneration and myonuclear accretion in the host myofiber.

## Different Niche Types in Bone and Muscle

Multiple subtypes of stem cell niches have been distinguished, i.e., simple niches, complex niches, and storage niches [17••]. The “simple niche” includes one type of cell interacting with another via cadherins, ephrins, connexins, and other cell–cell molecules [18, 19]. The network of interconnected mature osteocytes within a single osteon could be considered a simple niche. The “complex niche” includes different cell types that coordinately regulate each other to develop multiple cell behavior by means of niche regulatory signals [17••]. In bone, the niche comprised of (early) osteocytes, osteoclasts, osteoblasts, and their respective stem cells are a perfect example of a “complex niche.” Precise regulation of bone mass, and specifically bone architecture, requires complex interaction between these cells [20]. The “storage niche” includes cells that are maintained in the niche and quiescent until activated by external stimulation or signals [17••]. The MuSC niche is a typical example of a “storage niche.” Normally MuSCs are quiescent, but upon stimulation during injury or mechanical overload, they are activated, proliferate, and generate committed muscle precursors (myoblasts) or self-renewing daughters. Committed myoblasts differentiate and fuse together to restore damaged myofiber segments or fuse with the host myofiber and add new myonuclei to the myofiber, whereas self-renewing cells return to quiescence and repopulate the niche between the basal lamina and the sarcolemma.

## Cellular Niche Conditions for (Early) Osteocytes and Myoblasts

**Fig. 1:**
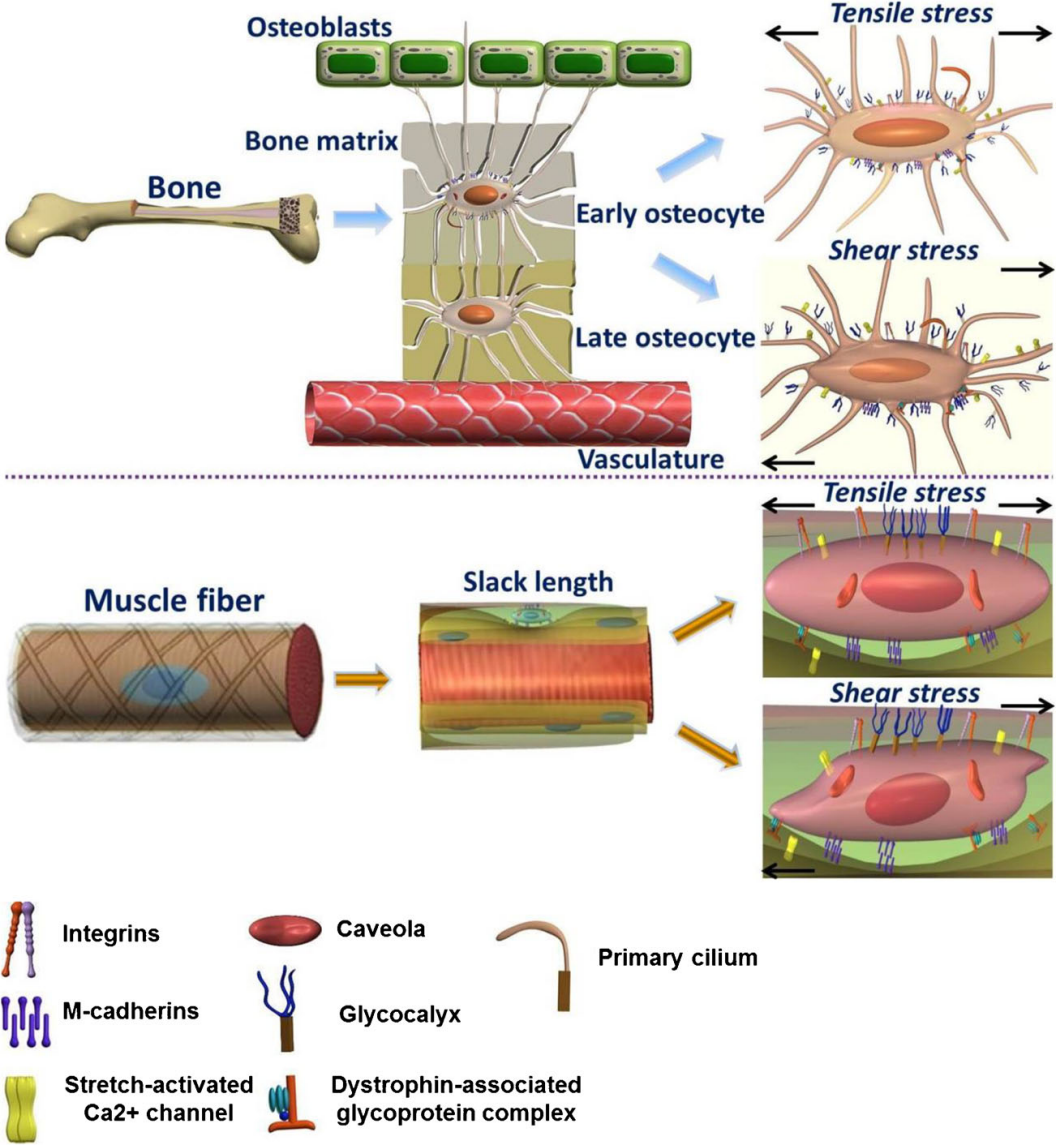
The process of how mechanical stimulation affects osteocytes and myoblasts within their native niche. Bone and muscle mass are regulated by many factors that include growth factors, hormones, nutrition, genetics, and, in particular, mechanical stimulation, e.g., tensile stress and shear stress upper panel shows the early and late osteocytes in their lacuna making contact with each other via their lamipodia extending through the canaliculi making contact with each other and with osteoblasts at the endosteum. Note that the late osteocyte is embedded within its calcified matrix while the matrix of the early osteocyte matrix is in a premature state of calcification. Due to the differences in calcification and stiffness, early osteocytes are likely subjected to pressure as well as tensile and shear deformations, while late osteocytes are subjected to fluid shear stress causing mechanical loading of transmembrane complexes and glycocalyx and presumably small local deformations within the plasma membrane at the cell body and lamipodia. MuSCs are enclosed between the sarcolemma of the host myofiber and the basal lamina and have transmembrane complexes via which the MuSC is connected to these structures. Upon shortening of the myofibers, MuSCs will be subjected to pressure and when myofibers are stretched, these cells will be strained and subjected to shear forces

An overview of the bone and MuSC niches is provided in Fig. 1.

### Cell Types of Bone

Bone contains osteogenic cells, periosteal fibroblasts, osteoblasts, pre-osteocytes, osteocytes, lining cells, and osteoclasts, which all interact [21]. Osteogenic cells can divide, proliferate, and differentiate into osteoblasts. Osteocytes are more responsive to mechanical loading than osteoblasts and periosteal fibroblasts and affect osteoblast proliferation and differentiation [21]. Osteocytes subjected to mechanical loading inhibit osteoclast formation and resorption by means of soluble factors [21]. The more deeply embedded osteocytes are only connected via their cell processes to neighboring osteocytes, but the most recently incorporated osteocytes (early osteocytes) are connected to neighboring osteocytes and to cells lining the bone surface. Moreover, some osteocytes processes oriented towards the bone surface appear to pass through the layer of lining cells, thereby establishing a direct contact between the osteocyte syncytium and blood vessels, or the extraosseus space. This latter intriguing observation by Kamioka et al. [22] suggests the existence of a signaling system between the bone marrow compartment and osteocytes. Bidirectional signaling between osteoclasts and osteocytes may exist through interaction between ephrinB2 on osteoclasts and EphB4 present on osteocytes [23]. Though purely hypothetical, osteoclast-to-osteocyte communication could be biologically relevant. In addition, the communication between osteoblasts and osteocytes has been investigated in vitro using the MLO-Y4 cell model under mechanical loading [24]. However, one limitation of the MLO-Y cells is that they only secrete low amounts of sclerostin. IDG-SW3 or OCY454 osteocytes have been employed as a model for early osteocytes. Moreover, OCY454 treated by mechanical loading do secrete a.o. sclerostin, suggesting that OCY454 cells are representing an early osteocyte model with the potential to sense mechanical loading [24, 25].

### Cell Types of Muscle (Table 1)

Skeletal muscle is composed of extremely long cells (i.e., myofibers) attached at both end to either tendon or bone. Myofibers are multinucleated post-mitotic cells consisting mainly of contractile filaments. In case of injury, the myofiber has the ability to regenerate by activation of MuSCs, also referred to as satellite cells. MuSCs are located on top of a myofiber enclosed between the basal lamina and the plasma membrane of the myofiber (i.e., sarcolemma; Fig. 1b). Within their niche, MuSCs are anchored to the sarcolemma of the myofiber by cadherins, while on their apical side they are anchored to the basal lamina of the endomysium via integrins, syndecans, and dystroglycans [27]. Outside the basal lamina, in the interstitial space, there are fibroblasts, adipocytes, endothelial cells, fibro-adipogenic precursors, and macrophages. In intact muscle, the communication between cells occurs via secretion of soluble signaling proteins. In the intact myofiber, the cells surrounding a MuSC are likely not in direct contact with this cell as this is prevented by the basal lamina. However, in an injured muscle with a ruptured basal lamina, MuSCs get into close vicinity and connect to these surrounding cells. In the beginning of muscle injury, immune cells, e.g., macrophages and monocytes, are important for regeneration after muscle injury. After immune cells are recruited, they infiltrate the muscle to get rid of necrotic tissue and secrete soluble factors to activate MuSCs, thereby building a transient local niche condition for these cells. Then MuSCs connect to immune cells via chemokines, and immune cells help MuSCs to escape from the basal lamina of myofibers and increase MuSC proliferation [27]. In muscle injury, muscle also includes a population of fibro-adipogenic progenitors (FAPs) which are characterized by PPARγ2. Normally, these cells are quiescent but they are activated upon injury. In adult tissue, FAPs are the main source of adipocytes and fibroblasts and important candidates for progenitors involved in muscle repair since there is very low homeostatic cell replacement in injured muscle. After acute damage, pro-differentiation signals (IL-6, Wnt family members, and insulin-like growth factors) may only be required in a short time, which is provided by, e.g., short-lived FAPs [30]. Stimulated FAPs also affect the MuSCs as they communicate with these cells via producing paracrine factors [30]. In addition, MuSCs are associated to neuromuscular junction (NMJ) degeneration and regeneration in response to denervation [31]. Possibly MuSCs modulate the extracellular niche condition of the myofiber via inhibiting the function of fibroblasts during skeletal muscle remodeling [31]. Myofiber type transition and reduction in muscle force generation capacity are highly correlated with NMJ disruption and effects of sciatic nerve transection are aggravated upon MuSC depletion [31]. The NMJ may affect the MuSC indirectly via its effect on the myofiber and the fibroblast but there may also be a direct interaction between the NMJ and the MuSCs [32]. Generally, MuSCs can interact with various cell types, e.g., macrophages, FAPs, regulatory T cells, endothelial cells, apelin, periostin, and oncostatin M [33].

**Table 1:** Molecular markers associated with myocytes, myoblasts, and muscle stem cells

Cell type	Molecular marker(s)	Reference
Myocyte	MyoD (+), myogenin (+), MHC (+), MCK(+)	[26]
MuSC	MyoD (+/−), Myf5 (+/−), Pax7 (+), β1 integrin (+) CXCR4 (+), α7 integrin (+), CD34 (+), VCam1 (+), c-Met (+), Mcad (+)	[27–29]
Myoblast	MyoD (+), Myf5 (+/−), Pax7 (+), β1 integrin(+), CXCR4 (+), α7 integrin (+), CD34 (+), VCam1 (+), and fibronectin (+)	[27–29]

## Chemical Niche Conditions for (Early) Osteocytes and Myoblasts

Here, we define chemical niche conditions as the non-cell-bound chemical cues that are consistently available in the extracellular environment of a specific cell and may affect the biological function of that cell. The most obvious example would be the chemical makeup of the ECM to which a cell is anchored (i.e., the available epitopes, defining the integrins which a cell can use to connect to the environment), but autocrine and paracrine soluble factors, and small molecules such as O_2_ and in case of bone also soluble calcium ions are good examples of chemical factors defining the niche conditions of bone and muscle cells.

Cells adhere to their substrate through integrins, which are frequently organized in focal adhesions. These focal adhesions serve as cytoskeletal anchor points connecting integrin clusters and actin filaments, which allow the transduction of both chemical and physical extracellular cues from the ECM across the membrane to the intracellular environment and the other way around. Integrins consist of 18 α and eight β subunits which assemble into 24 different receptors with an N-terminal head and extended C-terminal legs [34]. Their constitution depends on epitopes in the ECM. Thus, by understanding which integrins are present on a specific cell type, one can deduce which ECM proteins are available in the vicinity of a cell that drive cell behavior. Osteocytes contain β1 integrin [35], β3 integrin [36], and αvβ3 integrin [36]. In addition to integrins, unidentified molecules with a remarkable consistent length of ~ 100 nm connect the osteocyte cell membrane to the wall of the canaliculus. MuScs contain amongst others β1 integrin, α7 integrin, and β5 integrin. To the best of our knowledge, very little is known regarding the integrin mechanosensors in early osteocytes. Myoblasts also contain dystrophin, which is a cohesive protein linking actin filaments to other support proteins that reside on the sarcolemma. Dystroglycan is a dystrophin-associated glycoproteins, which anchors specifically to laminin in the basement membrane. In this way, the dystroglycan complex, which links the ECM to the intracellular actin filaments, provides structural integrity in muscle tissue. Key ligands in the ECM that bind to integrins and dystroglycans are fibronectin, laminin, and collagen.

Both osteocytes and MuSCs respond to growth factors and cytokines which have been reviewed extensively [37, 38] and alter intracellular signaling via tyrosine kinase receptors [39]. These factors may enter the ECM in an autocrine, paracrine, or endocrine manner. Other soluble factors that affect osteocyte and MuSC fate and function are metabolites, e.g., amino acids and vitamin D, and chemical elements, e.g., oxygen and its metabolites (i.e., nitrogen and oxygen-derived reactive oxygen species) [40].

### ECM Structural Components

The concept of ECM regulating cells is long-standing. The ECM provides a three-dimensional (3D) microenvironment for the cells. It is composed of collagens, fibronectin, elastin, laminin, glycosaminoglycans, and several other glycoproteins [41]. ECM provides many signaling molecules that modulate cell behavior and function, as well as triggers biological activities important in organ or tissue development and homeostasis [41]. Osteocytes are involved in the development and maintenance of the ECM in which they are embedded. For mature osteocytes, it is particularly important to precisely regulate the amount of calcification around the cell bodies and extensions. In healthy osteocytes there is a persistent matrix and fluid-filled gap of 50–80 nm between the calcified matrix and the cell membrane [42], which is of crucial importance for the transport of nutrients and oxygen through facilitated diffusion, and the transduction of mechanical signals. Early osteocytes, which are embedded in a collagen matrix that is not completely calcified, are also responding to mechanical loading [24]. In local mineralization regulated by osteocytes, there are several key proteins involved, e.g., DMP1, PHEX, MEPE, cathepsin K, and carbonic anhydrase [43, 44]. DMP1 is specifically expressed in the canaliculi of osteocytes and is secreted in the initial stages of mineralized matrix formation in bone [43]. PHEX is located on the membrane of osteoblasts and osteocytes and interacts extensively with DMP1 [43]. The small integrin-binding ligand N-linked glycoprotein family member MEPE is a marker of mature osteocytes and plays a role in the regulation of local matrix mineralization [43]. Cathepsin K and carbonic anhydrase are involved in osteocytic osteolysis, a process in which osteocytes remove calcified matrix in response to stimuli such as parathyroid hormone (PTH). The constituents of the matrix directly surrounding osteocytes has not been exactly defined, but proteins such as bone gamma-carboxyglutamic acid containing protein and noncollagenous proteins (i.e., osteopontin, osteocalcin, osteonectin, biglycan) have been shown to be present [45, 46].

The niche of MuSCs is created by ECM proteins, the vascular system, muscle residence cells, and muscle fibers [47]. This 3D structure surrounding each muscle fiber regulates cell–cell adhesion via integrin receptors increases the intermolecular binding to activate signaling pathway mechanisms in the myogenetic process and provides support for force transduction in or between cells [47]. So, it is necessary for bone and muscle cells to introduce ECM structural components. Glycocalyx contains glycoproteins and proteoglycans which are the common classes of glycans [48]. Glycocalyx may be connected with cytomembrane via glypicans or cytoskeleton via syndecans and extend into the ECM structure via glycosaminoglycan chains [48]. Growth factors can bind to the glycosaminoglycans and provide a store or facilitate the binding to the receptor. The glycocalyx is also involved in shear sensing [49].

### Signaling Molecules

Mechanosensitive osteocytes secrete several signaling molecules, i.e., nitric oxide (NO), prostaglandins, DMP1, PHEX, MEPE, FGF-23, BMPs, Wnts, osteonectin, osteocalcin, sclerostin, Lrp5, and RANKL [50]. After mechanical loading, osteocytes alter signaling molecule production, thereby affecting bone formation activity by osteoblasts and/or bone resorption activity by osteoclasts, and these cell types could be considered to exist in a complex niche [50]. However, many of these molecules may also affect the behavior of osteocytes in an autocrine or paracrine fashion, as in a simple niche. In addition, mechanically loaded myoblasts increase NO production, hepatocyte growth factor (HGF) gene expression [51], and proliferation-related gene expression [52]. These effects are important during exercise, when myofibers undergo substantial length changes by passive or active stretching, resulting in the opening of calcium channels and activation of integrin signaling pathways [51].

The relationship between bone and muscle might not only be determined by mechanical behavior but also by biochemical communication [53]. An overview of the details regarding cellular chemical signaling molecules is provided in Table 2.

**Table 2:** Cellular chemical signaling molecules

Molecule	Function	Relationship	References
NO	Modulation of osteoblast and osteoclast activity; produced after mechanical stimulation	L-arginine, NOS enzyme, molecular oxygen, NADPH, other cofactors	[54–56]
Ca^2+^	Regulation of cell or muscle contraction, cell motility, growth, proliferation	MTORC1, TrpV1, CaMKKα	[1]
Prostaglandins	Modulation of bone quantity and quality	EP2 receptor, fascin, COX-like enzyme, linker of nucleoskeleton, and cytoskeleton complex	[57]
BMPs	Induction of ectopic bone formation	Smads, TGF-β, activins	[58, 59]
Wnt/β-catenin	Modulation of osteoprogenitor proliferation; lineage selection	Axin, Dsh, GSK3, APC, PP2A, MYC, CCND1, LRP5	[60]
bFGF	Upregulation of eNOS expression; downregulation of RANKL expression	ERK1/2, c-Jun kinase (JNK), deltafosB, CREB, GEFs	[61, 62]
OPN	Regulation of bone cell differentiation; regulation of muscle size	Integrin-αv, integrin-β1, arginine-glycine-aspartate-containing glycoprotein	[63, 64]
OGN	Regulation of cell adhesion, proliferation, differentiation, migration in tissue development and repair	Alkaline phosphatase, collagen type I, β-catenin	[64]
IGF-1	Stimulation of muscle fiber growth; regulation of bone mass	PI3K/Akt/mTOR pathway, Murf1, MAFfbx	[64]
HGF	Stimulation of muscle cell regeneration; involved in bone formation and bone resorption	c-Met	[64]
VEGF	Regulation of oxidative metabolism of bone and muscle cells	Mechanical stimuli, hormones, growth factors, and transcription factors	[64, 65]
MAPK	Upregulation of eNOS expression; downregulation of RANKL	ERK1/2, c-Jun kinase (JNK), deltafosB, CREB, GEFs	[61, 62, 66]
Myostatin	Linkage of muscle atrophy with bone loss	Glucocorticoid	[67]
IL-4	Stimulation of bone cell proliferation; modulation of signaling pathways	MAPK, PI3K, mTOR	[68]
IL-6	Stimulation of insulin-stimulated glucose uptake; regulation of muscle fiber size; modulation of osteogenesis and bone resorption	AMPK	[64]

*BMP* bone morphogenetic protein, *bFGF* basic fibroblast growth factor, *OPN* osteopontin, *OGN* osteoglycin, *IGF-1* insulin-like growth factor-1, *HGF* hepatocyte growth factor, *VEGF* vascular endothelial growth factor, *MAPK* mitogen-activated protein kinase, *IL* interleukin, *NADPH* nicotinamide adenine dinucleotide phosphate, *mTORC1* mammalian target of rapamycin complex 1, *TrpV1* transient receptor potential cation channel subfamily V, *CaMKKα* Ca^2+^/calmodulin-dependent protein kinase kinase α, *EP2* prostaglandin E_2_ receptor 2, *COX* cyclooxygenase, *Smad* mothers against decapentaplegic homolog, *TGF-β* transforming growth factor-β, *Axin* axis inhibition protein, *Dsh* disheveled, *GSK3* glycogen synthase kinase 3, *APC* adenomatous polyposis coli, *PP2A* protein phosphatase 2, *MYC* myelocytomatosis, *CCND1* cyclin D1, *LRP5* lipoprotein receptor-related protein 5, *ERK* extracellular signal-regulated kinase, *CREB* cAMP-response-element binding protein, *AMPK* AMP-activated protein kinase

### Oxygen/metabolism

The oxygen tension is important for cell fate and function. It determines the oxidative metabolism and energy state. A low energy state (i.e., high ratio AMP/ATP) will activate adenosine monophosphate kinase (AMPK). Oxygen tension levels may be involved in the activation of hypoxia inducible factor 1α (*HIF1α*). At low oxygen level (i.e., hypoxia), *HIF1α* is not degraded and will regulate transcription of *HIF1A-* and/or *HIF1B-*responsive genes [69]. Activation of the *HIFα* signaling pathway in osteoblasts is important for osteogenesis and angiogenesis [70]. *HIFα* also regulates myoblast differentiation via activation of miR-210 transcription in myotubes [71]. Therefore, it is important to maintain intracellular oxygen homeostasis for bone and muscle cell function.

Hypoxia in environmental or clinical settings is potentially threatening tissue or organ oxygen homeostasis. In bone, hypoxia inhibits osteoblast growth and differentiation and strongly promotes osteoclast formation [72]. It has not been totally resolved whether and how oxygen sensing affects the function of osteocytes that remain in a low oxygen microenvironment. Oxygen sensing by the oxygen sensor prolyl hydroxylase-2 (PHD2) in osteocytes has been shown to decrease bone mass through epigenetic regulation of sclerostin, and targeting PHD2 results in an osteo-anabolic response associated with reduced bone resorption [73•]. Hypoxia is most likely the condition for the mature osteocytes and less so for the early, embedding osteocytes. In healthy bone, there is a persistent matrix and fluid-filled gap of 50–80 nm between the calcified matrix and the osteocyte cell membrane, which is of crucial importance for the transport of nutrients and oxygen through facilitated diffusion and the transduction of mechanical signals. In other words, not only mature osteocytes can be affected by many factors but also the early osteocytes which are embedded in a collagen matrix which is not completely calcified and which are relatively close to the bone surface. Early osteocytes are in a less calcified surrounding and likely less affected by hypoxia as oxygen tension is likely higher, however, in case of severe exercise or high altitude these cells may also sense hypoxia. Like in virtually all tissue, muscle is also affected by hypoxia via the HIF-1 signaling pathway [74]. Prolonged hypoxia does not promote angiogenesis in muscle [74]. However, hypoxia affects intracellular calcium concentration, which modulates muscle cell proliferation [75].

## Physical Niche Conditions of (Early) Osteocytes and Myoblasts

Mechanopresentation implies the presentation of mechanical load cues to be sensed by bone or muscle cells. When mechanical stimuli are applied to the cell surface or microenvironment, one or more ligands anchored on the cell surface to support mechanical force upon its application are required for mechanopresentation. Insoluble ligands such as integrins, syndecans, and dystroglycans are prerequisite, since soluble ligands cannot present mechanical cues. Mechanoreception is the process of attachment of the mechanopresenting ligand with the cell surface receptor that is exerted by mechanical load. The cell surface receptor is termed a cell mechanoreceptor, since it represents a molecule that senses and receives the mechanical load signal. This response may cause conformational changes of the cell surface-binding site of the ligand and receptor to change the bond properties. Mechanotransmission is executed by the mechanotransmitter, e.g., receptor and ligand. The mechanical load signal is transduced from the ligand receptor-binding site to the inside of the cell. Remarkably, the spreading of the mechanical load signal is not only limited to the mechanical force, since it does not only induce conformational changes of molecular signaling mechanisms, but it is also part of the mechanotransmission process, albeit its stimulatory effect on mechanotransduction that suggests that the mechanical stimuli are translated into biochemical signals. However, a model of tensegrity-based signaling in cells has been postulated that proposes that the cytoskeleton constitutes of a pre-stressed tensegrity structure allowing force transmission from the ECM onto the cytoskeleton and nucleus [76]. The forces exerted via the ECM cause the release of mRNA and ribosomes attached to the cytoskeleton and nuclear deformation or conformational changes of chromatin that affects gene transcription [77].

Mechanotransduction is either a local cell mechanosensing process or a whole cell mechanosensing process. Normally, a region of the receptor or its linked subunit structure undergoes conformational changes in a cell in response to a mechanical force waveform, which enables a biochemical event to occur in the cytoplasm. All the substances involved in the process of mechanotransduction are termed mechanotransducers, including focal adhesions, dystroglycan, glycocalyx, primary cilium, ion channels, and integrins [78, 79]. There are two pathways of the mechanotransduction process: (1) indirect mechanotransduction, which implies that the mechanical signal is transduced into a biochemical signal, and (2) direct mechanotransduction, which occurs via deformation of the cytoskeleton and nucleus. Mechanotransducers linking the ECM to the cytoskeleton are crucial for direct mechanotransduction [68].

### Matrix Stiffness

The importance of cell–ECM interactions in relaying mechanical signals has been well-known for two decades [76]. Stiffness is the mechanical property of ECM that cells can sense by the so-called stiffness sensing or rigidity sensing. The mechanism of stiffness sensing involves the contraction of myosin in the cell and is regulated by the mechanical properties of the ECM. Stiffness sensing occurs by myosin-dependent traction forces of cells and is likely affected by the number of focal adhesions and cell attachment to the ECM via high affinity integrin. Then actin stress fibers increase force transduction across the ECM–integrin–cytoskeleton connection [80]. Stiffness sensing also involves intracellular signaling. For example, Ca^2+^ concentration, which is regulated by mechanosensitive channels involved in stiffness sensing, as well as mechanosensitive adaptor proteins, may contribute not only to regulation of cell migration but also to substrate stiffness sensing [81]. Osteocyte-specific protein and osteoblast-specific gene expression have been investigated on substrates with different chemical composition and stiffness, as well as identical chemical composition but different stiffness. Osteocyte differentiation is highly increased on soft stiffness substrate at low seeding cell density [82].

MuSCs exhibit a strong regenerative ability in vivo, but this capacity is rapidly lost in vitro. The matrix or substrate stiffness is a potent regulator for MuSC behavior and fate in vitro [83]. MuSCs cultured on extremely thin hydrogel substrates of 2, 12, or 42 kPa stiffness mimic muscle elastic properties in vivo [83, 84]. Interestingly, MuSCs exhibit extensively muscle regeneration on a 12-kPa hydrogel substrate, but they do not spread and tend to differentiate on rigid matrix stiffness [84]. A cell sensing mechanical stimulus provides feedback and adjusts its adhesion strength and force accordingly via modifying cytoskeletal support and cell deformation, which enables constant communication between the microenvironment and the cell [85].

### External Mechanical Cues

Cells can be subjected to extracellular pressure, tensile force, and shear stress. Internal forces are exerted by traction forces generated by stress fibers of the cytoskeleton, which are attached to focal adhesions within the cell membrane. Pressure on bone affects mechanosensitive osteocytes embedded in the calcified bone matrix to orchestrate bone remodeling to maintain bone homeostasis under normal conditions [86]. Osteocyte-like MLO-Y4 cells can sense cyclic hydraulic pressure of 68 kPa at 0.5 Hz and respond via increasing intracellular Ca^2+^ concentration and changing microtubule organization. In addition, pressure increases gene expression of COX-2 and the RANKL/OPG ratio while decreasing apoptosis in osteocytes, suggesting that osteocytes play an important role in bone remodeling in vivo [86].

Intuitively one would not expect that osteocytes within bone are subjected to tensile forces and strains. However, bone loading by impact forces causes movement within the bone marrow [87]. Then early osteocytes within uncalcified ECM are also subjected to mechanical loading by movement of their surrounding matrix [24]. In addition to pressure and tensile stress, cells are subjected to shear stress when forces exerted onto the cell apex and base are oppositely directed. This occurs when forces are exerted to the apex or base via the ECM or when extracellular fluid or gelatinous matrix is displaced along the cell [88–90]. Due to anchoring and traction forces within the cytoskeleton, an opposite force will create a shear moment. In bone, early osteocytes are likely subject to shear loads, while late osteocytes are subjected to fluid shear stress around their cell processes. In vivo, shear stress in trabecular bone during whole bone loading produces micro-scale interaction between bone marrow and trabecular bone. It affects the distribution of mechanical stress and strain in bone marrow and its cellular components, e.g., hematopoietic stem cells, osteoclasts, pre-osteoclasts, and macrophages [91]. Tatsumi et al. [92•] developed a mouse model in which ~ 80% of the osteocytes are ablated upon injection with diphtheria toxin. The osteocyte-less mice are resistant to unloading-induced bone loss, confirming that viable osteocytes are essential producers of factors that activate osteoclasts in response to unloading. Osteocyte-less mice show an anabolic response to (re)loading of the bones, suggesting that the production of osteo-anabolic factors by mechanically stimulated osteocytes is not essential to achieve an increase in bone mass. This might indicate that osteoblasts or pre-osteocytes are mechanosensitive.

In contrast to bone, within muscle, tensile forces are continuously exerted onto the myofibers. Although it has never been shown that MuSCs in vivo or in situ within their niche are subjected to tensile strains and undergo strain deformations, it is highly likely that such deformations occur [93••]. As these cells are mechanically linked to the sarcolemma and the basal lamina, stretching and shortening of the myofiber will likely cause alignment of the MuSC with the myofiber and strain the cell. The stretching will cause elongation of the cytoskeleton together with the nucleus. Such length changes have been shown in cardiac myocytes and myofibers [94]. During movement, skeletal muscle is subjected actively or passively to large excursions by myofiber contractions (e.g., eccentric or concentric) or by external tensile forces, respectively [95]. Since muscle volume is constant [96], pressure within a muscle varies with its length. Moreover, due to myofascial connections between myofibers and between neighboring muscles, differences in length changes between myofibers and muscle induce shear forces onto myofibers and ECM. In addition, there exists an eccentric contraction can result in cytoskeleton and plasma membrane disruptions. In the beginning of eccentric contraction, fast glycolytic fibers fatigue. Then mitochondria of myocytes lose their calcium-buffering capacity as a result of their inability to regenerate ATP. Increased intracellular calcium activates calcium-activated lysosomal proteases, neutral proteases, and other cellular processes which are calcium mediated. It eventually causes myofiber damage [97]. Furthermore, the leakage of the enzymes and molecules may also affect the MuSCs [98].

## In Vitro Experiments

### How Valid Are In Vitro Experiments if Cellular Niche Is Indeed So Important?

Several transmembrane molecules and one organelle (i.e., cilium) have been implicated as mediators of mechanotransduction based on experiments with osteocytes cultured on flat substrates. Such experiments provide valuable insights into which molecules are produced by osteocytes in response to a mechanical stimulus, but they may be less useful for unraveling the mechanism behind osteocyte mechanotransduction. Although focal adhesion kinase is known to play a role in mechanosensing by osteocytes in vitro, osteocytes in situ do not form the large, sharply demarcated focal adhesions that they exhibit on stiff and flat surfaces.

Osteocytes in situ are surrounded by a specialized pericellular matrix and may employ a different subset of integrins for anchorage to their ECM than osteocytes on artificial matrices in vitro. Furthermore, a mechanical stimulus applied to flat adherent osteocytes in vitro predominantly affects the osteocyte cell body, while physical laws dictate that the extracellular fluid surrounding osteocytes in vivo/in situ only flows over the cell extensions. It is a fundamental paradox that the strains applied to whole bones may be as much as 30-fold lower than the strains necessary to trigger signaling in 2D cultured osteocytes. However, osteocytes on a flat surface spread out, and round non-adherent osteocytes are an order of magnitude more sensitive to a mechanical stimulus than flat osteocytes [99•]. Higher mechanosensitivity of cells with a more 3D morphology may thus provide part of the solution to the bone paradox. Taken together, it is still an enigma how osteocytes in situ transduce the minute mechanical stimuli that occur as a result of physical activity into a strong chemical response, and in vitro experiments may only provide limited clues to the mechanisms of osteocyte mechanotransduction.

**Fig. 2:**
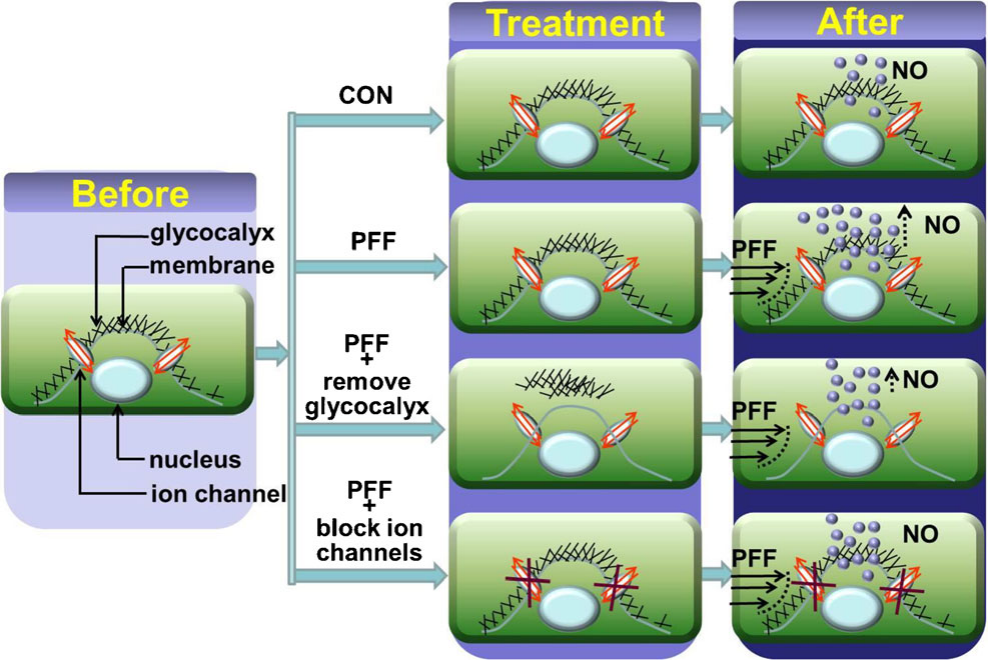
The experimental design to study interactions between the glycocalyx and ion channels of a cell in response to PFF. “Before” (pre-loading): with or without glycocalyx degradation and blocking of stretch-activated ion channels. “Treatment”: glycocalyx degradation and blocking of stretch-activated ion channels. “After”: NO production by C2C12 myoblasts in response to PFF with or without glycocalyx degradation and blocking stretch-activated ion channels. Myoblasts were seeded on a glass slide and cultured for 3 days, during which a glycocalyx was formed [15•]. Enzymatic removal of the glycocalyx from the cell surface was done by treatment with hyaluronic acid [15•, 100]. Stretch-activated ion channels were blocked by gadolinium chloride. Cells were stimulated by 1 h PFF, and NO production was measured after 0, 5, 10, 15, and 30 min [15•]. PFF, pulsating fluid flow

Stretch-activated ion channels and an intact glycocalyx are necessary to translate mechanical stimuli into a biochemical response [15•]. We studied the interaction between glycocalyx, stretch-activated ion channels, and mechanical loading in C2C12 myoblasts and found that removal of the glycocalyx eliminated pulsating fluid flow (PFF)-induced NO production, indicating that the glycocalyx is important for mechanosensing [15•] (Fig. 2). Moreover, NO production was ablated by blocking stretch-activated ion channels (Fig. 2).

### Unpublished Results

**Fig. 3:**
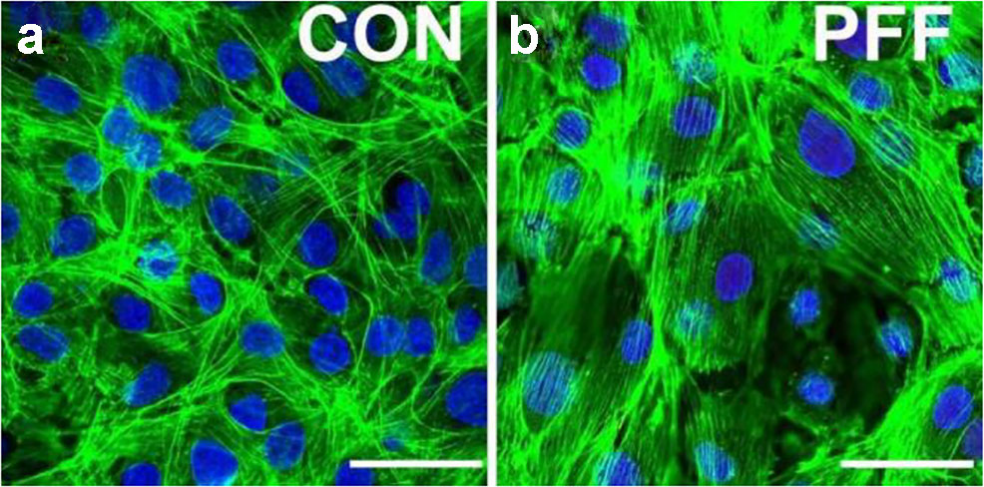
Changes in F-actin content and orientation with a bone cell subjected to PFF. MC3T3-E1 pre-osteoblasts were seeded on a glass slide, cultured for 3 days, treated with or without 1 h pulsating fluid flow (PFF), and fixed using 4% paraformaldehyde. F-actin was stained with rhodamine-phalloidin (green). Nuclei were stained with DAPI (blue). **a** Static control cell showing that F-actin stress fibers are woven into the complex network structure. **b** PFF-treated cell showing that F-actin stress fiber bundles are oriented and neatly organized in bundles. Magnification, × 100

To mimic the fluid shear stress that osteocytes are subjected to in vivo, we recently investigated the effects of PFF on cellular, cytoskeletal, and nuclear morphology in pre-osteoblasts in a 2D surrounding. Preliminary data show changes in key structures in bone cells that are crucial in determining cell morphology, i.e., F-actin, paxillin, α-tubulin, and integrin α5. One hour PFF increased F-actin in an area around the nucleus parallel to the long axis of the cell, which was consistent with findings by others [101]. Since at 24 h of culture the cell number was low and did not reach 100% confluency, we extended the cell culture time to 72 h. Then we observed that F-actin stress fibers were intricately interwoven into a network and appeared to cross-over each other to maintain cell morphology and function (Fig. 3a). Almost no stress fibers were visible around the nuclei. After 1 h PFF, the orientation and alignment of F-actin stress fiber bundles was almost homogeneous, with F-actin bundles running roughly parallel to the cell long axis orientation. In PFF-treated cells, the connections of F-actin bundles between cells appeared more simple and clear than in control cells (Fig. 3b). The F-actin stress fiber bundles were also clearly visible above the nuclei.

**Fig. 4:**
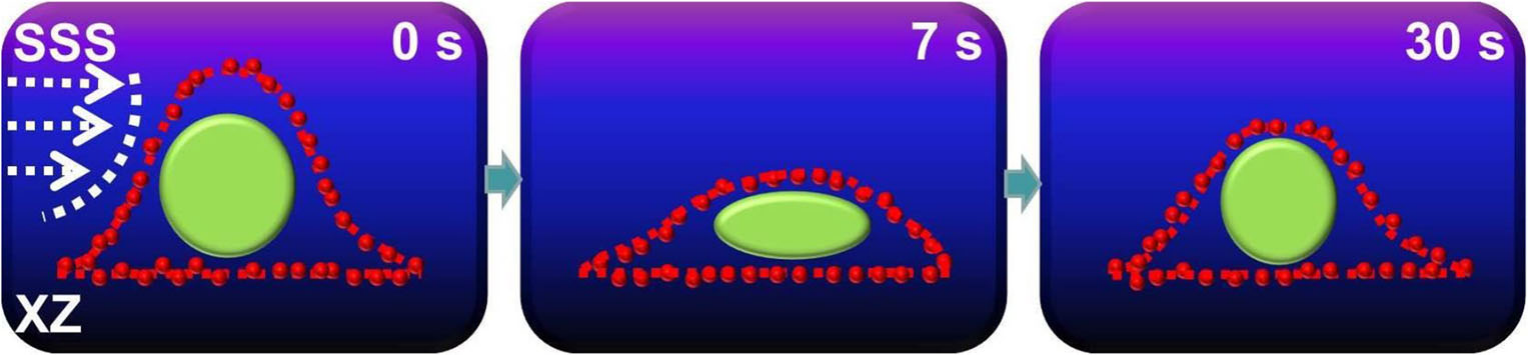
The changes in apex height of a live muscle cell subjected to static shear stress (SSS). This diagram is based on studies as described by Boers et al. [93••]. C2C12 myoblasts were seeded on a glass slide and cultured for 72 h [93••]. Cells were live-stained for F-actin (Sir-actin, red) and nucleic acid (Syto-9, green) for 4 h. Then the cell was subjected to SSS at 0.97 Pa for 3 min. Confocal microscopy was used to record the cross-sectional video of the cell (XZ direction). At 0 s, the longitudinal apex of cell and nucleus were highest. After SSS application for 7 s, the longitudinal apex of the cell and nucleus transiently collapsed. However, after 30 s, the C2C12 myoblast regained to a certain extent its original morphology

To determine cell structural changes, live cell imaging was performed on a C2C12 myoblast live-stained for the nucleus and cytoskeletal F-actin while being subjected to static shear stress (SSS) [93••]. The cell apex was highest at the start of SSS treatment (time = 0). After SSS application for 7 s, the apex of the cell transiently collapsed, while within 30 s, the myoblast regained its original shape ([93••], Fig. 4). Not only did the cell membrane and cytoplasm change the cell shape but also the nucleus showed substantial deformation as it was compressed. These data indicate that fluid shear stress will not only apply a horizontal force onto MuSCs and its mechanosensors but also increase pressure on the cell which results in membrane and cytoskeletal deformations and even nuclear deformations. In differentiated myoblasts, the glycocalyx is critically required for PFF-induced NO production [15•]. However, whether the glycocalyx plays a role in mechanosensing by MuSCs is unknown. Local stretching of the cell membrane will likely activate the stretch-activated calcium channels, which could elicit several signaling pathways. Further research is required to disentangle the functional role of the glycocalyx and the deformation of the cell membrane, cytoskeleton, and nucleus on signaling pathways and changes in gene expression.

**Fig. 5:**
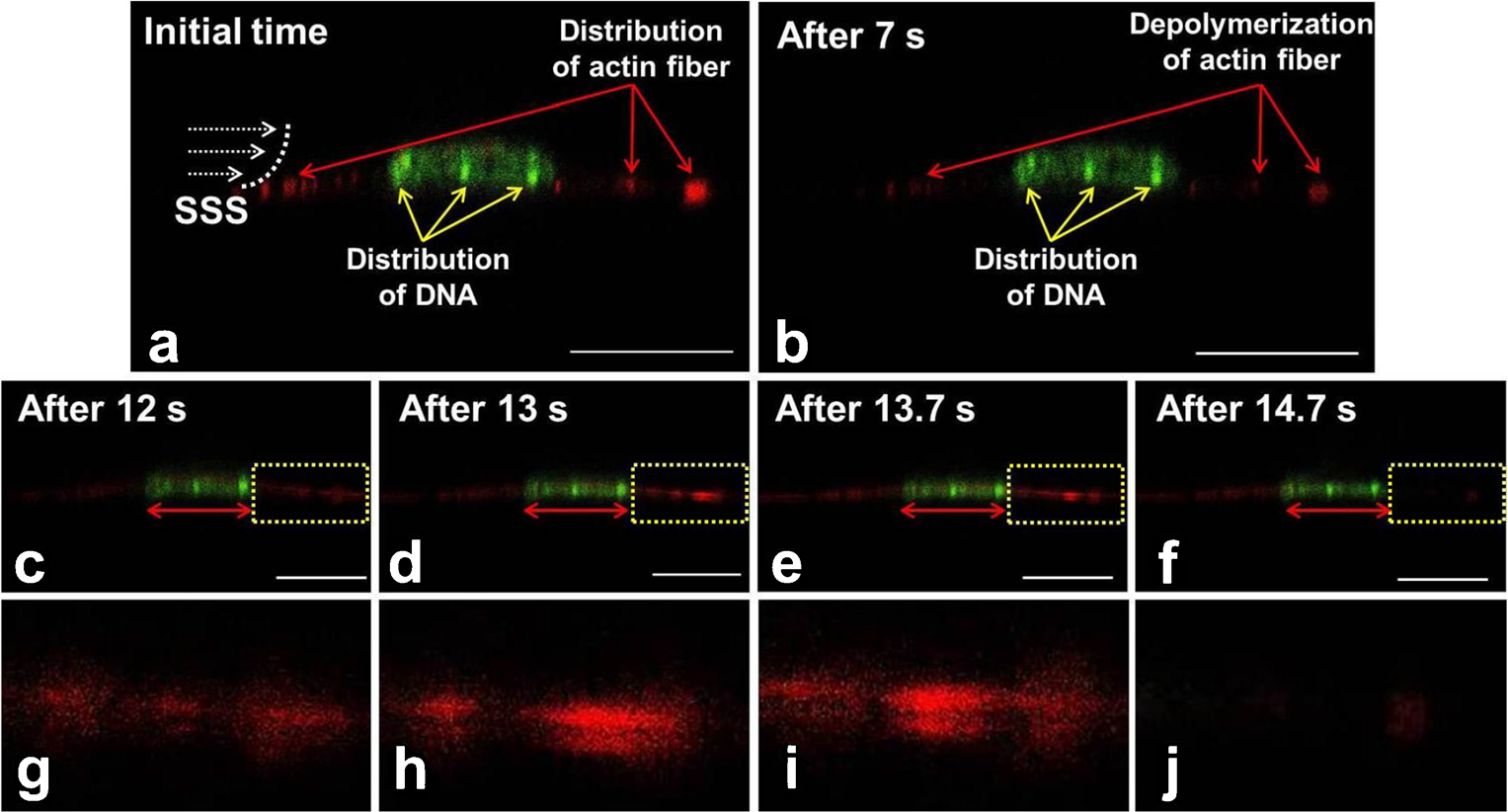
Live cell images of MC3T3-E1 pre-osteoblast deformation in response to static shear stress (SSS). Pre-osteoblasts were seeded on a glass slide and cultured for 24 h. Cells were stained for F-actin (Sir-actin, red) and nucleic acids (Syto-9, green) for 4 h. Then the cells were subjected to SSS (0.97 Pa) for 3 min [102]. Confocal microscopy was used to record a series of cross-sectional images of the cell (XZ direction). **a** Distribution of F-actin fiber and DNA before SSS treatment. **b** Distribution of F-actin fiber and DNA after SSS treatment for 7 s. **c** Structure of F-actin and nucleus in an SSS-treated cell after 12 s. **d** Structure of F-actin and nucleus in an SSS-treated cell after 13 s. **e** Structure of F-actin and nucleus in an SSS-treated cell after 13.7 s. **f** Structure of F-actin and nucleus within an SSS-treated cell after 14.7 s. **g**–**j** are the partial enlargement and correspond to the orange frames of **c**–**f**, respectively

To assess whether bone cells also show deformations in response to fluid shear stress, we subjected MC3T3-E1 pre-osteoblasts to SSS and show preliminary data of these experiments here. Before osteoblasts were subjected to SSS only little F-actin fiber fluorescent signal was observed (Fig. 5a). After 7 s SSS treatment, the F-actin signal was significantly reduced (Fig. 5b). After 12, 13, and 13.5 s, the F-actin signal increased and formed almost a straight line (Fig. 5c–d, g–i). After 14.7 s, the signal almost disappeared (Fig. 5f, j). Interestingly, some nuclear changes were seen, i.e., with the time-lapse of mechanical loading, the DNA distribution fluctuated, and the F-actin fluorescence intensity slightly weakened as the nucleus was slightly compressed, showing that mechanical loading affects cell structure and (de)polymerization of F-actin. Fluid shear stress may also affect deformation of early osteocytes, which are embedded in a collagen matrix which is not completely calcified. It is expected that such deformations will become less when osteocytes mature and their niche becomes calcified. However, osteocytes are highly mechanosensitive and small local deformations may be sufficient to elicit a response. Taken together, cells significantly change their behavior in response to external mechanical stimuli. Future investigations will address how SSS or PFF will deform cells with or without glycocalyx.

## Effects of Aging and Disease on the Osteocyte and MuSC Niche

During aging, osteocyte and MuSC numbers are reduced in bone and skeletal muscle, respectively [cf. 103–106]. Aging does not alter osteocyte mechanosensitivity, but ER stress probably contributes to reduce the anabolic response to mechanical stimuli in old osteocytes, while it might not play a role in the blunted response of aged muscle to anabolic stimuli [107, 108]. Osteocytes located in smaller lacunae will experience lower strains than those in larger lacunae and will respond less to the same mechanical loading than those in larger lacunae, indicating that smaller lacunae such as found in aged bones are a potential causative factor for the reduced bone mechanoresponsiveness seen with aging [109–111]. These studies will have pivotal implications for a better understanding of the role of osteocyte lacunar morphology on functional bone adaptation and the disturbed bone remodeling process at old age. In aging muscle, the MuSC ability to proliferate is reduced and is accompanied by fibrosis which increases the stiffness of the niche [93••]. This enhanced niche stiffness may alter gene expression, since the MuSCs will sense this increase in stiffness via traction forces. However, the fibrosis will also alter the external loads applied to the cells. The impact of aging-associated fibrosis as well as this impaired regenerative capacity of the MuSC in aging muscle needs further investigation.

Reciprocal communication between cell and microenvironment plays a crucial role in maintaining normal cell or tissue function. The conditions that block these feedback loops, either by influencing intracellular or extracellular physical force distribution, cellular mechanosensing, or signal transduction, can result in various clinical phenotypes [79]. In the past years, more and more evidence has been provided that fine-tuning the feedback between the cells and their physical microenvironment is crucial to the cell structure and function, ranging from adhesion, spreading, and viability, to differentiation and proliferation [79]. Interference with cellular and microenvironmental mechanotransduction processes may thus lead to diseases affecting various tissues and/or organs [79]. Studying the underlying mechanisms of these diseases may lead to adopt new therapeutic strategies, such as improvement of tissue engineering design and enhancement of biomaterials, and also provide us with more opportunities to learn, know, and understand physicochemical niche conditions, mechanosensing, and mechanobiology in normal cells and physiology [79].

## Conclusions

Osteocyte and MuSC function is dependent on their native niche characteristics, but the cells also affect their own niche. It is a two-way street. The process of mechanotransduction can be divided into distinct stages. First, osteoblasts, osteocytes, or MuSCs subjected to PFF secrete signal molecules such as NO and prostaglandins. Second, integrins form clusters with intracellular anchoring complexes which relay signaling molecules into the cell from the ECM. Third, signaling molecules affect the arrangement or polymerization of actin and tubulin. Fourth, actin and tubulin connect the plasma membrane to the nucleus membrane, which might play a role in determining the cell and nucleus morphology and volume. Finally, changes in cell and nucleus morphology and volume cause upregulation of gene and protein expression. In the future, it needs to be determined whether and which changes in cell morphology drive osteogenic differentiation, as well as the underlying mechanism. A combination of in vivo and in vitro studies is necessary to determine the effects of physicochemical niche conditions on both bone and muscle cells, microenvironmental mechanosensing, and mechanotransduction. Targeting cell and microenvironment is expected to promote bone and muscle regeneration and maintain homeostasis in response to mechanical stimulation in healthy individuals and in patients suffering from disease or injury.
